# A simplified and effective off‐axis Winston–Lutz for single‐isocenter multi‐target SRS

**DOI:** 10.1002/acm2.13816

**Published:** 2022-11-24

**Authors:** Anton Eagle, Mike Tallhamer, Justin Keener, Sarah Geneser

**Affiliations:** ^1^ Centura Health Longmont United Hospital Hope Cancer Care Center Longmont Colorado USA; ^2^ Centura Health Parker Adventist Hospital Parker Colorado USA; ^3^ Centura Health Littleton Adventist Hospital Littleton Colorado USA; ^4^ Department of Radiation Oncology University of Washington Seattle Washington USA

**Keywords:** SIMT, SRS, Winston–Lutz

## Abstract

**Purpose:**

To safely perform single‐iso multi‐target (SIMT) stereotactic radiosurgery (SRS), clinics must demonstrate SRS delivery accuracy for off‐axis targets. The traditional Winston–Lutz (W–L) was widely adopted because it provides a simple and accurate solution for testing radiation‐isocenter coincidence that uses a static target, enables testing arbitrary treatment angles, and does not require expensive commercial phantoms. The current noncommercial tests are cumbersome and insufficiently accurate. For an off‐axis Winston–Lutz (OAWL) test, one must design MLC fields centered on off‐axis targets. Unfortunately, because MLC leaf‐interfaces are often misaligned with the target center, accomplishing this presents a nontrivial geometry problem that has not been previously solved in the literature. We present a solution for evaluating SIMT SRS accuracy that provides a straightforward method for creating OAWL test fields and offers all the benefits of the standard W–L test.

**Methods:**

We have developed a method to use any gantry, table, and initial collimator angles to create OAWL fields. This method calculates a series of nested coordinate transformations that produce a small collimator angle adjustment to align the MLC and create a symmetric field around an off‐axis target.

**Results:**

For an 8 cm off‐axis target, the described method yields OAWL results within 0.07 mm of standard isocentric W–L results. Our six most recent isocentric W–L tests show max and mean errors of 0.59 and 0.37 mm, respectively. For six runs of our proposed OAWL test, the average max and mean errors are 0.66 and 0.40 mm, respectively.

**Conclusion:**

This method accurately evaluates SRS delivery accuracy for off‐axis distances that span the majority of a typical human brain for a centered SIMT arc. We have made this method publicly available, so that physicists can employ it within their clinics, foregoing the need for expensive phantoms and improving access to the state‐of‐the‐art SIMT SRS technique.

## 1 INTRODUCTION

### 1.1 Single‐iso multi‐target SRS

Stereotactic radiosurgery (SRS) provides a substantial improvement in local control for patients with multiple brain metastases.^1^, ^2^, ^3^ For decades, patients presenting with multiple SRS targets required separate treatments for every individual target, each with a separate isocenter, requiring substantial time to set up and deliver.^4^ More recently, technological advancements allow the simultaneous treatment of multiple SRS targets with a single isocenter. The delivery efficiency gains of single‐iso multiple‐target SRS (SIMT SRS) have resulted in a steady increase in the demand for the method.

One of the significant challenges to the widespread adoption of SIMT SRS is the lack of a simple and effective quality assurance test to demonstrate targeting accuracy at distances that span the majority of an average adult human brain (16.7 × 14.0 × 9.3 cm^3^).^5^ An SRS arc centered amongst multiple small targets can potentially result in off‐axis targets as great as ∼8.4 cm from isocenter. The ability to show that these targets can be treated with an accuracy similar to that seen with isocentric targets would facilitate more rapid adoption of SIMT SRS.

Ensuring that isocentric Winston–Lutz (W–L) deviations are less than 1 mm is the widely accepted standard for SRS treatments.^6^ However, for off‐axis positions, targeting accuracy will necessarily degrade with increased off‐axis distance.^7^, ^8^, ^9^, ^10^ This decreased accuracy is due to the increased effect of gantry, collimator, and couch angular errors as a function of [*off‐axis‐distance*] × SIN(*θ*) for any angular error, *θ*. This concept is further discussed in Appendix B.

To safely treat multiple off‐axis targets using linac‐based SIMT SRS, we need a test of off‐axis targeting accuracy that is as useful as the standard W–L isocentric test. To match the utility of the isocentric W–L, this test should ideally have the following characteristics:

- utilize a static unmoving target,
- provide physicists an efficient means of creating test fields using their current treatment planning system (TPS) tools,
- allow for the use of a variety of gantry, couch, and collimator angles that mimic the actual SRS treatment delivery, and
- not rely on the use of expensive third‐party phantoms or software.

Previous works on this topic have either used nonstationary targets,^7^, ^8^ resulted in deviations greater than 1 mm for the required off‐axis distances,^7^, ^8^, ^9^, ^10^ or involved the purchase of expensive commercial phantoms and software.^9^, ^11^ These previous works are summarized in Section 4.

### 1.2 Challenges

**FIGURE 1:**
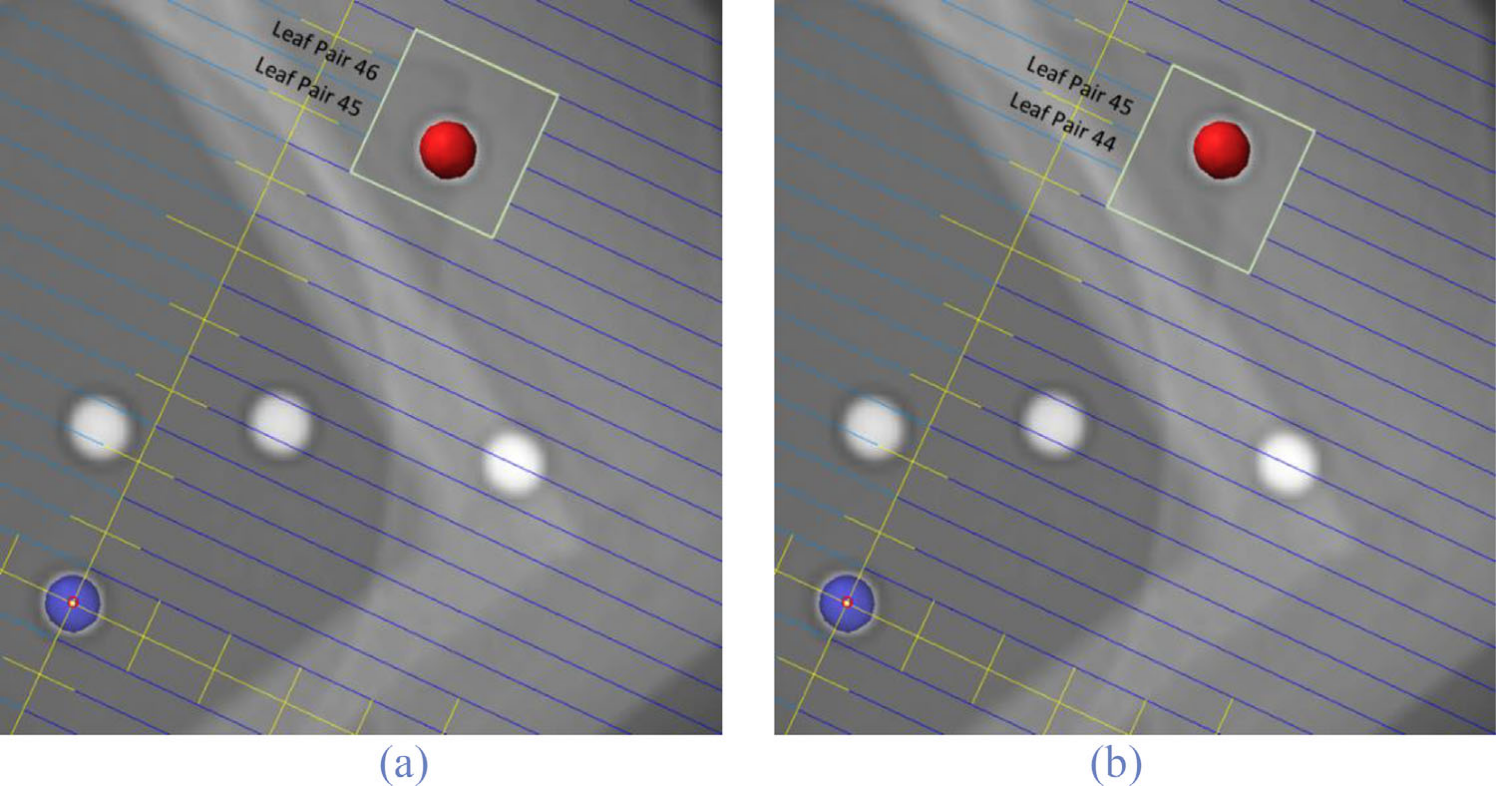
These images depict the beam's eye views (BEVs) for test fields with arbitrarily selected gantry, collimator, and couch angles of 335°, 335°, and 35°, respectively. The phantom is aligned with target 1 (depicted in blue) at isocenter. Note that no leaf‐interfaces are centered on the off‐axis target (red), making it infeasible to create a symmetric field about this target using the MLCs. Parts (a) and (b) depict attempts to create a test field using the closest MLC leaves, which highlights the inability to center a field on the off‐axis target for these treatment angles.

The primary challenge in developing an off‐axis Winston–Lutz (OAWL) test that meets the previously listed criteria resides not with the design of the phantom, but rather with the design of the test fields. Designing an appropriate phantom is relatively simple, requiring only visible landmarks to setup the phantom and an off‐axis target of known position. In contrast, there are several difficulties regarding the creation of fields for OAWL tests. The primary challenge is that the fields must be designed using MLCs to create a symmetric field centered on the off‐axis target. For an off‐axis target with arbitrary gantry, collimator, and couch angles, the MLC aperture will typically not be centered in the *Y*‐axis (Figure 1) because the inter‐leaf interfaces will not align with the center of the target. Note: In this manuscript, the term *inter‐leaf interface* refers to the space between adjacent leaves, and for the sake of brevity, this term will henceforth be shortened to *leaf‐interface*.

Figure 1a,b shows a test field with arbitrarily chosen treatment angles, specifically: gantry angle 335°, collimator angle 335°, and couch angle 35°. Note that because of leaf‐interface misalignment, for this combination of treatment angles, it is not possible to create a test field that is centered on the target in the *Y*‐axis using the MLCs.

To solve this, we need to align a leaf‐interface with the center of the target. The needed alignment only occurs for specific combinations of gantry, collimator, and couch angles. These angles can be found by trial and error in a TPS or solved for exactly using coordinate transformations. Determining these angles using brute‐force trial and error in a TPS requires significant time and effort and is only as accurate as the measuring tools in the TPS. Solving for these angles exactly comprises a nontrivial geometry problem that has not been presented in prior literature.

### 1.3 Proposed solution

Our solution hinges on the observation that given the geometry described in Figure 1, one can simply rotate the collimator a small amount to bring an MLC leaf‐interface into alignment with the center of the off‐axis target. The appropriate collimator angle correction, for any arbitrary combination of couch, gantry, and initial collimator angles, can be easily calculated to center a symmetric MLC field on any off‐axis target. This generalized solution enables the use of any static phantom of known geometry, with any TPS system, and satisfies all the characteristics of a simple and easy OAWL test as described in Section 1.1. Additionally, we have made freely available to all physicists a spreadsheet demonstrating this solution, and the link to an online repository where this can be found is listed in Appendix C.

## 2 MATERIALS AND METHODS

To create an MLC field using arbitrary treatment angles centered on an off‐axis target, we perform sequential coordinate transformations to calculate the precise position of the target in the beam's eye view (BEV). An additional correction to the collimator angle is then needed to align an MLC leaf‐interface to the center of the target. Matrices using standard perspective projections or affine coordinate transformations with associated scaling can be used to determine the BEV coordinates of the off‐axis target. However, simple trigonometry can also be used, which enables the creation of an easy‐to‐follow spreadsheet. We therefore use sine–cosine functions to sequentially calculate the needed transformations.

### 2.1 Define the coordinate system

Using the Varian Standard coordinate system, let *X_phan_*, *Y_phan_*, and *Z_phan_* be the target's three‐dimensional coordinates relative to isocenter, where the *X*‐axis is oriented right–left (patient's right‐left if head‐first supine), the *Y*‐axis is anterior–posterior, and the *Z*‐axis is inferior–superior.

For a given OAWL test field with corresponding TPS‐defined table (couch), gantry, and collimator rotations, labeled as *Ɵ_t_*_,_ *_TPS_*, *Ɵ_g_*_,_ *_TPS_*, and *Ɵ_c_*_,_ *_TPS_*, respectively, we ultimately solve for the adjusted collimator angle, *Ɵ_c_*_,_ *_final_*, that brings the MLC leaf‐interface into alignment with the center of the target.

First, we solve for the target position within the BEV. To do this, we begin with the target's position (in the TPS coordinate system) with gantry, couch, and collimator each at 0° and sequentially solve for the change in the target position that results from each rotation.

**FIGURE 2:**
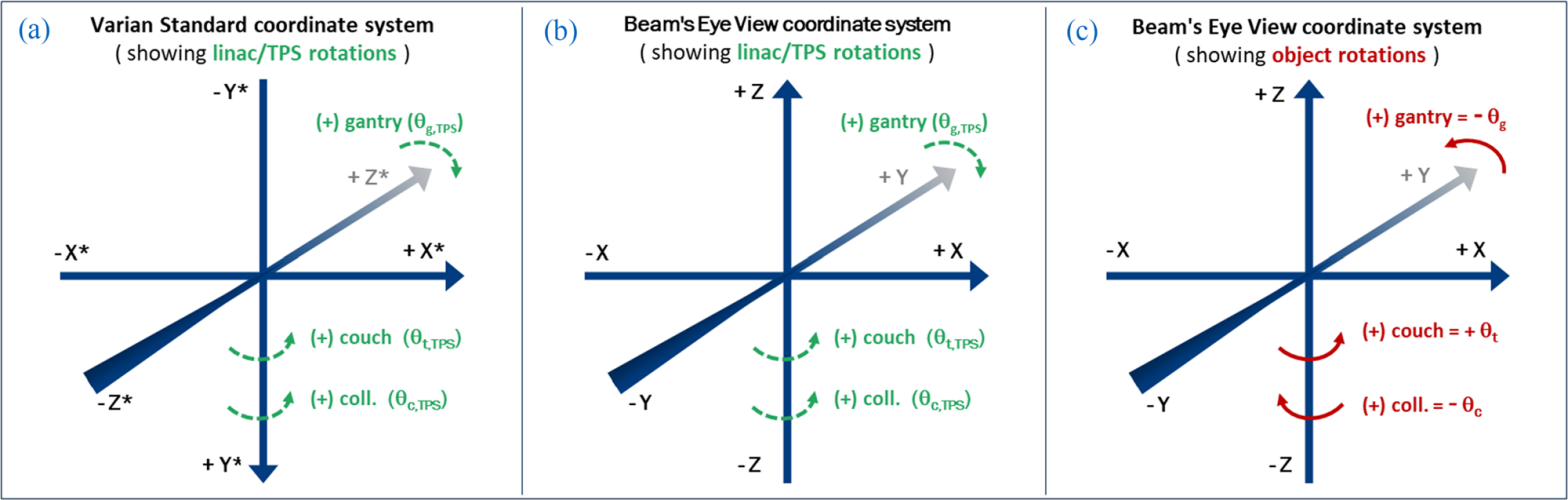
Part (a) depicts our treatment planning system (TPS) coordinate system (X*, Y*, Z*) and the sign of TPS axis rotations. Part (b) depicts the beam's eye view (BEV) coordinate system (*X*, *Y*, *Z*) and its rotation configuration. Part (c) shows the apparent object rotations (in the BEV) due to axis rotations and also shows the resultant sign for these rotations used in the coordinate transformations.

Before applying the coordinate transformations, one must determine the directionality (sign) of the rotations involved. This depends on the handedness of the coordinate system. In our case, the TPS coordinate system is right‐handed. Thus, correctly identifying the sign of a rotation involves the right‐hand rule, where clockwise rotations (as observed with the axis pointing away from the viewer) are denoted as positive angles. The BEV system, which we ultimately use for our final field parameters, is inherently right‐handed with a two‐dimensional (2D) *X*–*Y* plane and an unseen *Z*‐axis pointing up toward the viewer. It is important to note that the angular readout values denoted in the TPS and linac coordinate systems do not necessarily conform to the right‐hand rule and may differ depending on the specific coordinate systems employed by each clinic. Moreover, the BEV coordinate system does not necessarily align with the TPS or linac coordinate systems. For our TPS, increases in couch rotation values correspond to positive rotations in the BEV coordinate system, whereas increases in both collimator and gantry values correspond to negative BEV rotations as shown in Figure 2. Using our coordinate transformation nomenclature, this gives *Ɵ_t_* = *Ɵ_t_*_,_ *_TPS_*, *Ɵ_c_* = −*Ɵ_c_*_,_ *_TPS_*, and *Ɵ_g_* = −*Ɵ_g_*_,_ *_TPS_*. Note that these may vary for other TPS systems.

Importantly, the rotations shown in Figure 2c refer to the rotation of the object within the coordinate system, not the rotations of the axes themselves. For the couch, the object rotates with the axis. However, for the gantry and collimator, the apparent object rotations within the BEV will be the opposite of the axis rotation, as the object is stationary for those, which is why the directionality appears to flip for those axes.

### 2.2 Apply coordinate transformations

Now that we have determined the correct sign for the rotations, we perform the actual coordinate transformations. Starting with a table (couch) rotation *Ɵ_t_*, the target's *X*, *Y*, and *Z* position becomes

(1) $$X_{t}=X_{phan}\times \cos\left(\theta_{t}\right)-Z_{phan}\times \sin\left(\theta_{t}\right)$$

(2) $$Y_{t}=Y_{phan}$$

(3) $$Z_{t}=X_{phan}\times \sin\left(\theta_{t}\right)+Z_{phan}\times \cos\left(\theta_{t}\right)$$

After a subsequent gantry rotation, $\theta_{g}$, the position is

(4) $$X_{g}=X_{t}\times \cos\left(\theta_{g}\right)-Y_{t}\times \sin\left(\theta_{g}\right)$$

(5) $$Y_{g}=X_{t}\times \sin\left(\theta_{g}\right)+Y_{t}\times \cos\left(\theta_{g}\right)$$

(6) $$Z_{g}=Z_{t}$$

After the collimator rotation, $\theta_{c}$, the coordinates are

(7) $$X_{c}=X_{g}\times \cos\left(\theta_{c}\right)-Z_{g}\times \sin\left(\theta_{c}\right)$$

(8) $$Y_{c}=Y_{g}$$

(9) $$Z_{c}=X_{g}\times \sin\left(\theta_{c}\right)+Z_{g}\times \cos\left(\theta_{c}\right)$$

Translating these results to the BEV coordinate system, the new variables are *X_BEV_*_,_ *_target_* = *X_c_* and *Y_BEV_*_,_ *_target_* = *Z_c_*.

Next, we correct for the magnification factor of the target. In the previous transformations, the *Y_c_* coordinate (Equation 8) determines the final source to target distance. The BEV geometry is defined at isocenter using a source‐to‐image distance of 100 cm, and we must therefore project the target onto an image plane at this distance. Thus, the BEV 2D coordinates resulting from this magnification are

(10) $$X_{\mathrm{BEV},\mathrm{targ}\mathrm{et}}=X_{c}\times 100/\left(100+Y_{c}\right)$$

(11) $$Y_{\mathrm{BEV},\mathrm{targ}\mathrm{et}}=Z_{c}\times 100/\left(100+Y_{c}\right)$$

### 2.3 Calculate collimator angle corrections

To obtain a square field aperture centered on the target, the leaf‐interface misalignment in the *Y*‐axis direction in the BEV can be corrected by applying an additional collimator rotation. There are many possible collimator angles that will suffice, so long as the resultant *Y* coordinate aligns with a leaf‐interface. In the following, we solve for the two closest leaf‐interfaces that always yield the two closest collimator angles, lying to either side of the initial collimator angle. Note that if the initial collimator angle is near the physical limit such that one solution lies beyond that limit, one can always choose the other.

To find these new collimator angles, we note that the hypotenuse (*H*) of the triangle formed by *X* and *Y* coordinates stays constant with collimator rotation and use the Pythagorean theorem to calculate the new angle and new *X* coordinate.

**FIGURE 3:**
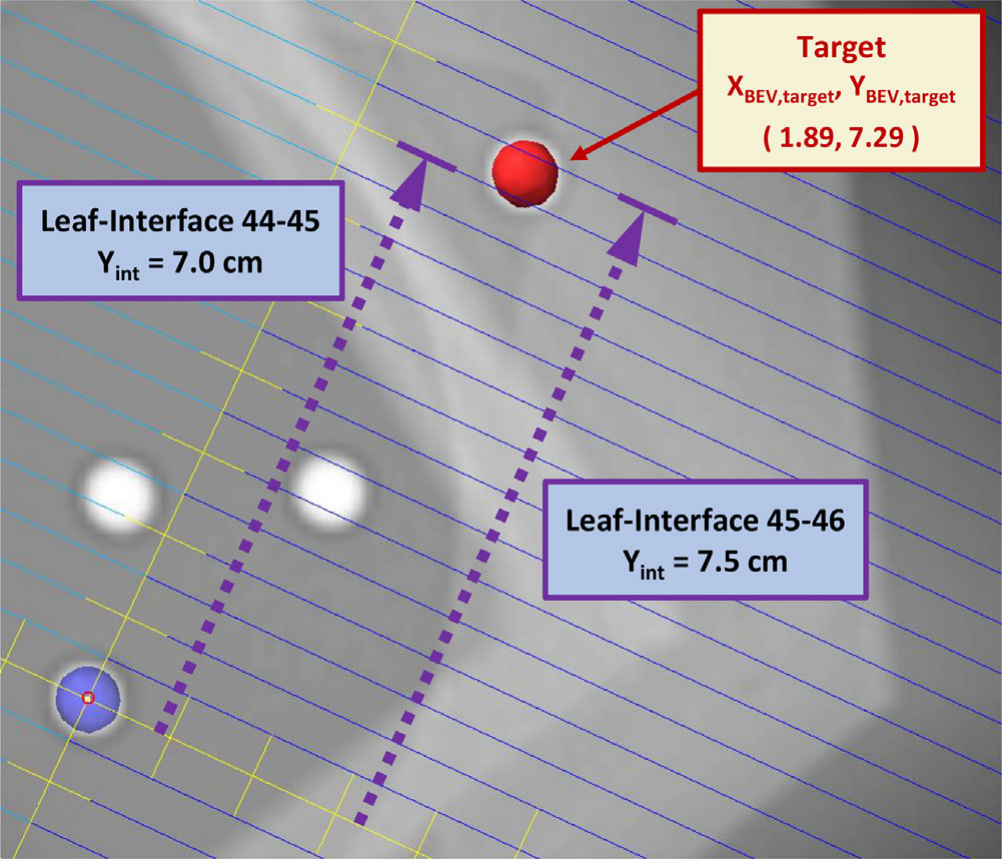
Target position, *X_BEV_*_,_ *_target_* and *Y_BEV_*_,_ *_target_*, with the *Y*‐coordinate (*Y_int_*) of the two nearest leaf‐interfaces

Thus, for a given *X_BEV_*_,_ *_target_*_,_ and *Y_BEV_*_,_ *_target_*, we select a nearby leaf‐interface to rotate into alignment. *Y_int_* is then the *Y*‐axis distance to the selected leaf‐interface (in the BEV) as shown in Figure 3, which becomes our new *Y_BEV_*_,_ *_final_* (Equation 12). Next, we calculate the new *X_BEV_*_,_ *_final_* using the Pythagorean theorem (Equation 13). Note that the result of the Pythagorean is inherently positive and does not retain the sign of the *X* coordinate, so the correct sign for the *X* coordinate must be manually carried over from *X_BEV_*_,_ *_target_*. Next, we calculate the amount of collimator angle correction, *Ɵ_c_*_,_ *_corr_*, needed to make the *Y*‐coordinate in the BEV equal to this distance to the leaf‐interface (Equation 14), and apply that correction to obtain the final collimator angle *Ɵ_c_*_,_ *_final_* (Equation 15):

(12) $$Y_{BEV,final}=Y_{int}$$

(13) $$X_{\mathrm{BEV},\mathrm{final}}=\mathrm{sign}\left(X_{\mathrm{BEV},\mathrm{targ}\mathrm{et}}\right)\sqrt{H^{2}-{Y_{\mathrm{int}}}^{2}}$$

where $\mathrm{sign}\left(x\right)=\frac{x}{\left|x\right|}$ and is employed to carry over the sign from *X_BEV_*_,_ *_target_*, as the result of the Pythagorean is inherently positive:

(14) $$\begin{array}{ccc} & & \theta_{c,\mathrm{corr}}=\arctan\left(Y_{\mathrm{BEV},\mathrm{final}}/Y_{\mathrm{BEV},\mathrm{final}}X_{\mathrm{BEV},\mathrm{final}}X_{\mathrm{BEV},\mathrm{final}}\right)\\ & & -\arctan\left(Y_{\mathrm{BEV},\mathrm{targ}\mathrm{et}}/Y_{\mathrm{BEV},\mathrm{targ}\mathrm{et}}X_{\mathrm{BEV},\mathrm{targ}\mathrm{et}}X_{\mathrm{BEV},\mathrm{targ}\mathrm{et}}\right) \end{array}$$

(15) $$\theta_{c,\mathrm{final}}=\theta_{c}-\theta_{c,\mathrm{corr}}$$

Applying this method to the field shown in Figure 3, with initial collimator angle 335° and *Y_BEV_*_,_ *_target_* = 7.29 cm, the closest leaf‐interface is between leaves 44 and 45, or between leaves 45 and 46. Aligning the first leaf‐pair, with *Y_int_* = 7.0 cm = *Y_BEV_*_,_ *_final_*, results in a *Ɵ_c_*_,_ *_final_* of 342.1° and *X_BEV_*_,_ *_final_* of 2.78 cm. Aligning to the second leaf pair, with *Y_int_* = 7.5 cm = *Y_BEV_*_,_ *_final_*, produces *Ɵ_c_*_,_ *_final_* = 325.8° and *X_BEV_*_,_ *_final_* = 0.70 cm. These examples are fully detailed in Appendix A.

**FIGURE 4:**
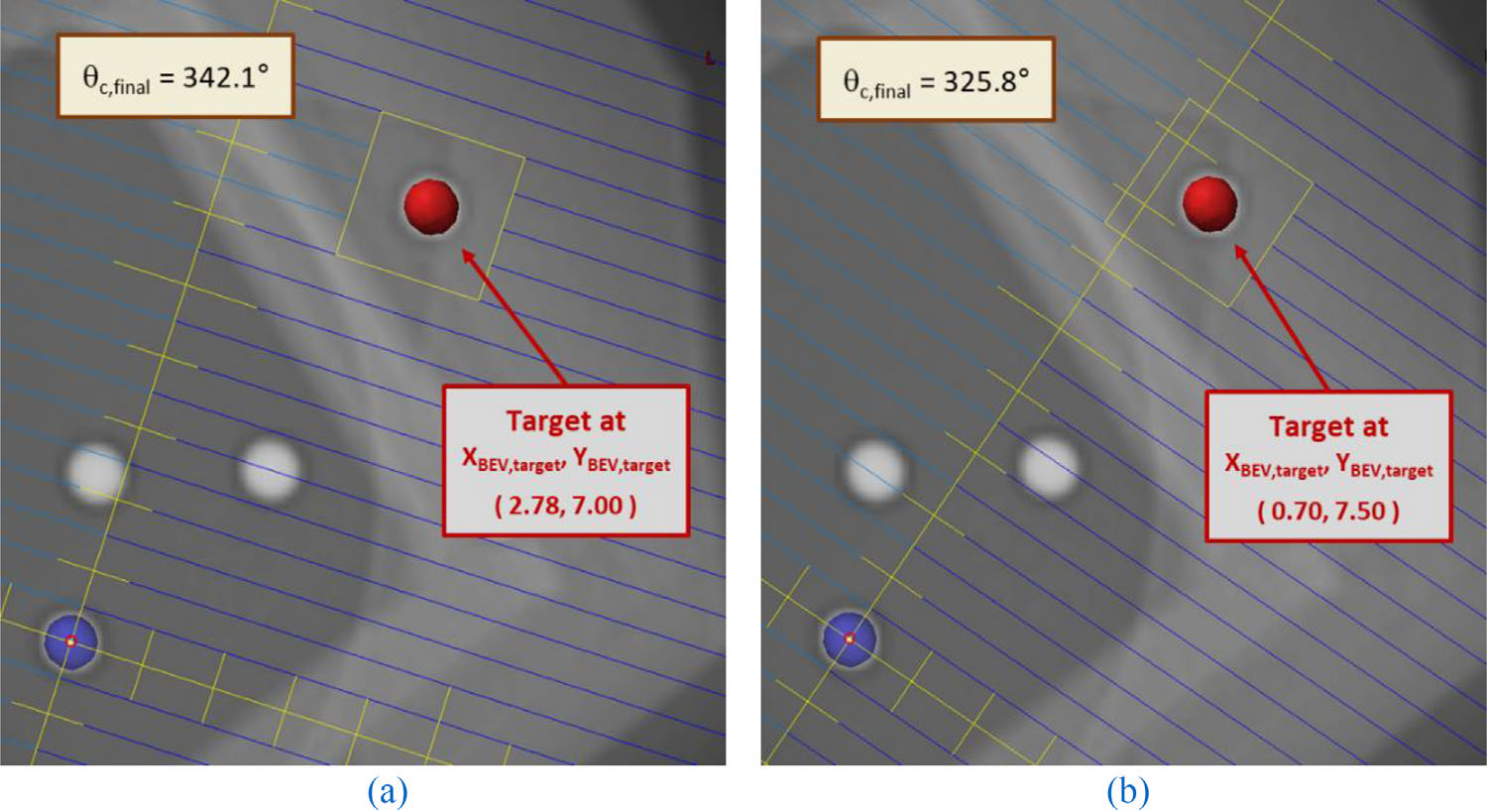
The beam's eye view (BEV) of symmetric off‐axis Winston–Lutz (OAWL) fields for the off‐axis target with collimator angles of 342.1° (a) aligning leaf‐interface 44–45, and 325.8° (b) aligning leaf‐interface 45–46, respectively

Applying the previous corrections to collimator angles and noting that the BEV—*X* coordinate (*X_BEV_*_,_ *_final_*) gives the precise MLC position of the center of the target in the *X*‐axis, we get the following MLC shapes (Figure 4) for a 2 × 2 cm^2^ centered on the target, aligning the leaf‐interface at *Y_int_* = 7.0 cm or *Y_int_* = 7.5 cm, respectively. We obtain a centered aperture in the *X* direction by manually entering *X_BEV_*_,_ *_final_* ±1 cm for MLC leaf values in the TPS. This gives us MLC values of 1.78 and 3.78 cm for Figure 4a, and MLC values of −0.3 and 1.70 cm for Figure 4b, for the four leaf‐pairs bracketing the target. Because our MLC leaves are 0.5 cm thick, we set these MLC values for two leaves on either side of the target center, resulting in a field symmetrically centered in both *X* and *Y*. Using a different field size will require different MLC settings.

### 2.4 Off‐axis Winston–Lutz test plan

All tests of the proposed solution were performed on a Varian TrueBeam with Millenium‐120 MLC using the VisionRT calibration cube; a 15 × 15 × 15 cm^3^ phantom containing 5 7.5 mm diameter alumina–ceramic spheres. One marker is positioned in the center of the cube with the other four positioned as shown in Table 1. The coordinates shown in Table 1 are positional offsets from the cube center given in the Varian Standard coordinate system where the *X*‐axis is oriented right–left (patient's right–left if head‐first supine), the *Y*‐axis is anterior–posterior, and the *Z*‐axis is inferior–superior. It is worth noting that this phantom was chosen simply because it was already available in our clinic. Our proposed method is in no way limited to this particular phantom. Indeed, any phantom with an off‐axis target at a location sufficient to test the desired treatment off‐axis distances will suffice for our solution.

**TABLE 1:** Absolute and relative (to target 1) coordinates of ball‐targets in VRT cube phantom

	Absolute coordinates (cm)			Coordinates relative to target 1 (cm)			
Target	*X*	*Y*	*Z*	*X*	*Y*	*Z*	Total off‐axis distance from target 1 (cm)
1	−2.5	3.0	−1.0	–	–	–	–
2	−1.5	1.5	1.0	1.0	−1.5	2.0	2.7
3	0.0	0.0	0.0	2.5	−3.0	1.0	4.0
4	2.0	−1.0	−2.0	4.5	−4.0	−1.0	6.1
5	3.0	−2.0	2.0	5.5	−5.0	3.0	8.0

To test off‐axis setup accuracy with the largest possible off‐axis distance, we used target 1 for the setup isocenter, and target 5 as the treatment target. Table 1 also shows the coordinates relative to target 1 and the resultant off‐axis distances.

The total off‐axis distance of 8.0 cm ensures that we can test a range that covers the majority of a typical adult brain with an SRS arc where the isocenter is properly chosen to be centered amongst an array of targets. Admittedly, there will be uncertainty in the reported position of internal targets in any phantom. However, we do not rely on the accuracy of the reported positions, as we can directly measure the positions in the CT dataset. Note that regardless of phantom, it is important to verify the off‐axis positions in the TPS. All test plans were created using Varian Eclipse v16.1, with the TPS setup using the Varian Standard coordinate system. The phantom was scanned using a Philips Brilliance‐16 Big‐Bore CT, using the O‐MAR artifact reduction reconstruction option. The OAWL images were obtained with the onboard MV EPID panel on the TrueBeam. The OAWL results were analyzed using Varian‐Mobius DoseLab Pro v6.7, though many other software applications could be used as the analysis is identical to the standard W–L test.

Using the OAWL method described herein, we created a plan for various combinations of gantry, couch, and collimator angles. The angles were chosen to span the clinically useful range and are shown in Table 2. We also evaluated combinations of oblique angles in our preliminary investigations but noted that these fields consistently showed lower errors than the angles included later and, thus, were deemed unnecessary. Importantly, the determination of which angles to test will depend on the performance of each individual linear accelerator and should be chosen accordingly. Additionally, the exact adjusted collimator angle needed will also depend on your MLC model, linac model, and phantom used.

**TABLE 2:** Off‐axis Winston–Lutz gantry, couch, and collimator angles

Field no.	Gantry	Couch	Collimator
1	0	0	1.6
2	270	0	358.1
3	90	0	3.6
4	180	0	356.2
5	0	0	85.1
6	0	0	274.1
7	0	90	4.1
8	0	270	4.1

The initial positioning of the phantom was accomplished with VisionRT surface guidance. Using VisionRT only resulted in a quicker setup, and the use of a surface guidance system is in no way required. A setup CBCT was used for final positioning. Phantom setup is simple, straightforward, and mimics the procedures used to setup patients clinically. Performing the OAWL test does not take significantly more time than performing an isocentric W–L, with only an additional 5–10 min spent with phantom alignment and CBCT registration.

## 3 RESULTS

Using our novel OAWL method, we obtained six sets of eight W–L images (one image for each of the fields described in Table 2). Each OAWL setup was performed independently. Table 3 shows the deviations for all OAWL tests and, for comparison, the deviations for traditional isocentric W–L tests from the same linear accelerator. The results shown were obtained using the Varian DoseLab W–L application. *Note that the mean maximum deviation for the OAWL is only 0.07 mm greater than the isocentric test and is well within the maximum allowable SRS tolerance of 1.0 mm* ^6^ *and also within the more conservative and widely accepted SRS tolerance of 0.75 mm*.^12^ The term “delta” in Table 3 refers to the 2D offset of the target in the W–L image as reported by the DoseLab W–L application.

**TABLE 3:** Off‐axis Winston–Lutz (OAWL) results

OAWL run	Maximum delta (mm)	Maximum total 3D delta (mm)	Mean total delta (mm)
OAWL 1	0.67	0.67	0.34
OAWL 2	0.55	0.58	0.34
OAWL 3	0.63	0.65	0.40
OAWL 4	0.66	0.72	0.52
OAWL 5	0.65	0.66	0.43
OAWL 6	0.66	0.69	0.39
Mean OAWL deviations	0.64	0.66	0.40
Mean isocentric W–L deviations (for comparison)	0.57	0.59	0.37

Abbreviations: 3D, three‐dimensional; W–L, Winston–Lutz.

In Table 4, we summarize our results as compared to the previous works on this topic.

**TABLE 4:** Summary of off‐axis Winston–Lutz results

Study	Off‐axis dist. (cm)	Max error (mm)	Mean error (mm)	Off‐axis dist. (cm)	Max error (mm)	Mean error (mm)
Gao et al.^7^	4	∼1.1	Unreported	5	∼1.7	Unreported
Gao et al.^8^	3	0.80	0.54	6	1.02	0.77
Poder et al.^9^	3	0.88	0.49	6	0.97	0.57
Gilson^10^				4.1	1.03	0.32–0.54
Eagle et al. (2022)				8.0	0.72	0.40

Our W–L images were acquired using a standard Varian TrueBeam with no special equipment or configuration. When run on other TrueBeams within our hospital system, we see similar results.

## 4 DISCUSSION

The primary goal in designing this methodology is to reduce the sources of error in an OAWL test to match (as much as possible) the sources of error in the traditional isocentric W–L test. We minimize errors introduced during plan design by calculating the precise BEV position of the off‐axis target instead of relying on measurements in the TPS. Additionally, we eliminate errors due to couch motion as, once initially setup, the couch position is not changed in our methodology. As noted previously, additional uncertainties are introduced that stem from potential errors in the internal target positions. These uncertainties will exist for any phantom with off‐axis targets but are largely mitigated by directly measuring the target positions in the TPS. In Appendix B, we provide an estimation of the additional uncertainties that the OAWL test introduces compared to the standard W–L.

Additionally, the difference between our current work and the previous works detailed earlier is not just one of improved accuracy. More notably, this new OAWL procedure represents an improved and simplified methodology. Some previous attempts at an OAWL test moved the target to the off‐axis position, adding couch motion errors to a test that is designed to isolate only isocentricity errors.^7^ Other attempts used fields that were not centered on the targets (similar to those depicted in Figure 1) and manually corrected the W–L results using those estimated offsets.^11^ Lastly, some used an expensive commercial phantom and software that only work with specific pre‐calculated field geometries.^9^, ^11^ *In comparison, our work uses a stationary phantom, calculates centered MLC fields for any phantom at any set of treatment angles, does not require a commercial phantom, and can be analyzed with any software used for an isocentric Winston–Lutz test*.

Our results show that we now have an OAWL test that can demonstrate the required accuracy for off‐axis distances that span the majority of a typical adult human brain when treating with an SIMT SRS arc. Importantly, our method directly calculates the parameters needed to create centered test‐fields, enabling traditional W–L image analyses. This allows for the use of all well‐established commercial W–L software tools, eliminating more error‐prone manual measurements.

## 5 CONCLUSION

For the first time in the published literature, it is possible to obtain OAWL results below the accepted and more conservative SRS tolerance of 0.75 mm using off‐axis distances that span the majority of an adult human brain. By reducing the sources of error, we achieve comparable results to the isocentric W–L test, with maximum deviation and mean error within 0.1 mm of the isocentric test.

The results of an OAWL test can inform a clinic how far off‐axis they can safely and accurately treat SRS targets using a single isocenter. Achieving results that are submillimeter (and even less than 0.75 mm) ensures that traditional SRS PTV margins may still be used when transitioning from single‐target to multi‐target SRS. Treating SIMT without performing an OAWL test would not be recommended, as deviations in angle readout will magnify the translational errors the further the target is off‐axis. The tolerances listed in TG‐142, for gantry, couch, and collimator readout, may need to be tightened to maintain stereotactic accuracy for SIMT, and we intend to investigate the appropriateness of those published values in a future work.

Lastly, we feel that it is important to provide this solution to the entire medical physics community to help facilitate the widespread adoption of SIMT SRS. To this end, we provide spreadsheets that compute solutions for our method in a publicly available repository for use by the entire medical physics community. See Appendix C for a link to this file.

## AUTHOR CONTRIBUTIONS

Anton Eagle designed the proposed solution, performed the data analysis, and wrote the manuscript. Michael Tallhamer contributed to the solution design, contributed to the data analysis, and contributed to manuscript editing. Justin Keener contributed to the solution design, contributed to the data analysis, and contributed to manuscript editing. Sarah Geneser contributed to writing the manuscript, significantly assisted in editing, and contributed to final manuscript approval.

## CONFLICT OF INTEREST

The authors declare no conflict of interest.

## Data Availability

All Winston–Lutz results were analyzed using Varian Mobius DoseLab, and the reports showing each and every W–L image are available, immediately, upon request.

## Appendix A

In the following example, we show the steps required to calculate the two collimator angles discussed in Sections 2.2 and 2.3 and shown in Figure 3. Although there exist multiple possible solutions for aligning a leaf‐interface with the target via the methodology described herein, we chose the leaf‐interface nearest to the target in order to limit the angular correction required to properly center the field.

First, we detail the calculation of the position of the target center in the beam's eye view (BEV), as shown in Equations (1) through (11). Starting with the initial treatment planning system (TPS) coordinate (target 5 in Table 1) of *X_phan_* = 5.5 cm, *Y_phan_* = −5.0 cm, and *Z_phan_* = 3.0 cm, and using TPS angles of couch = 35°, gantry = 335°, and collimator = 335°, we first note that our coordinate system requires a sign change for the gantry and collimator angles (as described in Section 2.1). Thus, for use in Equations (1)–(11), we have *Ɵ_t_* = +35, *Ɵ_c_* = −335, and *Ɵ_g_* = −335.

Using these values for theta, Equations (1)–(3) give us the coordinates after applying the couch (table) rotation:

(A1)
$$\begin{array}{ccc} X_{t} & = & X_{\mathrm{phan}}\times \cos\left(\theta_{t}\right)-Z_{\mathrm{phan}}\times \sin\left(\theta_{t}\right)\\ & = & \left(5.5\right)\left(0.819\right)-\left(3.0\right)\left(0.574\right)=2.78\mathrm{cm} \end{array}$$

(A2)
$$Y_{t}=Y_{phan}=-5.0\mathrm{cm}$$

(A3)
$$\begin{array}{ccc} Z_{t} & = & X_{\mathrm{phan}}\times \sin\left(\theta_{t}\right)+Z_{\mathrm{phan}}\times \cos\left(\theta_{t}\right)\\ & = & \left(5.5\right)\left(0.574\right)+\left(3.0\right)\left(0.819\right)=5.62\mathrm{cm} \end{array}$$

Using Equations (4)–(6), we get the following after applying the gantry rotation:

(A4)
$$\begin{array}{ccc} X_{g} & = & X_{t}\times \cos\left(\theta_{g}\right)-Y_{t}\times \sin\left(\theta_{g}\right)\\ & = & \left(2.78\right)\left(0.906\right)-\left(-5.0\right)\left(0.423\right)\\ & = & 4.64\mathrm{cm} \end{array}$$

(A5)
$$\begin{array}{ccc} Y_{g} & = & X_{t}\times \sin\left(\theta_{g}\right)+Y_{t}\times \cos\left(\theta_{g}\right)\\ & = & \left(2.78\right)\left(0.423\right)+\left(-5.0\right)\left(0.906\right)\\ & = & -3.35\mathrm{cm} \end{array}$$

(A6)
$$Z_{g}=Z_{t}=5.62\mathrm{cm}$$

Then, using Equations (7)–(9) to apply the collimator rotation, we have

(A7)
$$\begin{array}{ccc} X_{c} & = & X_{g}\times \cos\left(\theta_{c}\right)-Z_{g}\times \sin\left(\theta_{c}\right)\\ & & =\left(4.64\right)\left(0.906\right)-\left(5.61\right)\left(0.423\right)\\ & = & 1.83\mathrm{cm} \end{array}$$

(A8)
$$Y_{c}=Y_{g}=-3.35\mathrm{cm}$$

(A9)
$$\begin{array}{ccc} Z_{c} & = & X_{g}\times \sin\left(\theta_{c}\right)+Z_{g}\times \cos\left(\theta_{c}\right)\\ & = & \left(4.64\right)\left(0.423\right)+\left(5.61\right)\left(0.906\right)\\ & = & 7.04\mathrm{cm} \end{array}$$

Lastly, using Equations (10) and (11) gives us the coordinates in the BEV and applies the magnification factor resulting from the *Y_c_* coordinate:

(A10)
$$\begin{array}{ccc} X_{\mathrm{BEV},\mathrm{targ}\mathrm{et}} & = & X_{c}\times 100/\left(100+Y_{c}\right)\\ & = & \left(1.83\right)\left(\frac{100}{100+\left(-3.35\right)}\right)\\ & = & \left(1.83\right)\left(1.035\right)\\ & = & 1.89\mathrm{cm} \end{array}$$

(A11)
$$\begin{array}{ccc} Y_{\mathrm{BEV},\mathrm{targ}\mathrm{et}} & = & Z_{c}\times 100/\left(100+Y_{c}\right)\\ & = & \left(7.05\right)\left(\frac{100}{100+\left(-3.35\right)}\right)\\ & = & \left(7.05\right)\left(1.035\right)\\ & = & 7.29\mathrm{cm} \end{array}$$

In Figure 2, we can see that the leaf‐interface between leaf‐pairs 45 and 46, or 44 and 45, are closest to the initial target *Y* position, which in this case is *Y* = 7.29 cm. Leaf‐interface 44–45 is at *Y* = 7.0 cm, and leaf‐interface 45–46 is at *Y* = 7.5 cm. We can obtain corrected collimator angles that result in *Y_int_* coordinates that match the previous leaf‐interfaces. To find these, we note that the hypotenuse of the triangle formed by BEV—*X* and *Y* coordinates stays constant with collimator rotation and in this example is equal to 7.53 cm.

Thus, for leaf‐interface 44–45, we have

(A12)
$$Y_{\mathrm{int}}=7.0\mathrm{cm}=Y_{\mathrm{BeV},\mathrm{final}}$$

(A13)
$$\begin{array}{ccc} X_{\mathrm{BEV},\mathrm{final}} & = & \mathrm{sign}\left(X_{\mathrm{BEV},\mathrm{targ}\mathrm{et}}\right)\sqrt{H^{2}-Y^{2}}\\ & = & +\sqrt{7.53^{2}-7.0^{2}}\\ & = & +\sqrt{7.70}\\ & = & +2.78\mathrm{cm} \end{array}$$

This gives a collimator angle correction of

(A14)
$$\begin{array}{ccc} \mathrm{Col}l_{\mathrm{corr}} & = & \arctan\left(7.0/7.02.782.78\right)\\ & & -\arctan\left(7.29/7.291.891.89\right)\\ & = & 68.3^{\circ }-75.4^{\circ }\\ & = & -7.1^{\circ } \end{array}$$

The new collimator angle is

(A15)
$$\theta_{c,\mathrm{final}}=\mathrm{Col}l_{\mathrm{orig}}-\mathrm{Col}l_{\mathrm{corr}}=335^{\circ }-\left(-7.1^{\circ }\right)=342.1^{\circ }$$

For leaf‐interface 45–46, we have

(A16)
$$Y_{\mathrm{int}}=7.5\mathrm{cm}=Y_{\mathrm{BEV},\mathrm{final}}$$

(A17)
$$\begin{array}{ccc} X_{\mathrm{BEV},\mathrm{final}} & = & \mathrm{sign}\left(X_{\mathrm{BEV},\mathrm{targ}\mathrm{et}}\right)\sqrt{H^{2}-Y^{2}}\\ & = & +\sqrt{7.53^{2}-7.5^{2}}\\ & = & +\sqrt{0.45}=+0.67\mathrm{cm} \end{array}$$

(A18)
$$\begin{array}{ccc} \mathrm{Col}l_{\mathrm{corr}} & = & \arctan\left(7.5/7.50.670.67\right)\\ & & -\;\arctan\left(7.29/7.291.891.89\right)\\ & = & 84.9^{\circ }-75.4^{\circ }=+9.5^{\circ } \end{array}$$

(A19)
$$\theta_{c,\mathrm{final}}=\mathrm{Col}l_{\mathrm{orig}}-\mathrm{Col}l_{\mathrm{corr}}=335^{\circ }-\left(9.5^{\circ }\right)=325.5^{\circ }$$

The previous results may vary slightly from the final results shown in Figure 2, due to rounding at the individual steps, and these values are only shown for demonstration purposes. Additionally, note that any resultant collimator angle that is greater than 360° represents an angle in the TPS (or on the linac) that has moved past the 0° point and should be corrected by subtracting 360°.

To obtain a centered field, decide how large you want your field to be in increments equal to the thickness of your MLC leaves. For the *X*‐axis, set the MLC positions to offset from the target center by half the width of the field. Thus, the leaves will be set at positions equal to *X_BEV_*_,_ *_final_* ± [field size]/2, for a number of leaves such that the field height is equal to the width. In our example, we choose a field size of 2 × 2 cm^2^.

For the field with *θ_C_*_,_ *_final_* = 324.1°, this gives us MLC values of 1.78 and 3.78. For the field with *θ_C_*_,_ *_final_* = 325.5°, this gives us MLC values of −0.33 and 1.67 cm. Because our MLC leaves are 0.5 cm thick, we set these MLC values for 2 leaves on either side of the target center, resulting in a symmetrically centered field in both *X* and *Y*. Again, note that if using a spreadsheet, these values will be slightly more accurate, as they will not apply rounding at every step.

## Appendix B

In the following, we estimate the uncertainties in this procedure. The uncertainties due to voxelization during simulation CT are not included, as these exist in any radiation therapy plan, and exist equally for this test and the traditional isocentric Winston–Lutz (W–L) test. Similarly, uncertainties due to pixelization in the W–L image exist equally for the traditional isocentric test and our off‐axis test. Instead, we will focus on the additional sources of error that might affect our proposed solution that do not exist for the isocentric test.

Given that both the TPS and the linear accelerator provide couch positions to the nearest 0.01 cm (coordinates in cm to 2 decimal places), all positions will be accurate to within ±0.005 cm. These errors exist equally for off‐axis and isocentric tests and thus do not represent an additional source of error. However, for this test, we introduce additional error in BEV position due to changes in gantry, couch, and collimator angles. These angles are shown to only 1 decimal place, resulting in an uncertainty of ±0.05°. The maximum translational error resulting from this angular uncertainty is [*off‐axis distance*] × SIN(0.05), which, in our case, is equal to 8.0 cm × 0.000873 = 0.0070 cm = 0.070 mm. Adding all three angular uncertainties in quadrature gives us 0.121 mm. Both the calculated value and the linac readout are independent, so these too sum in quadrature. All sources of uncertainty due to significant digits in angle readout total to 0.17 mm and are shown in Table 5. This value represents a worst case scenario, and the actual error will typically be much smaller.

**TABLE 5:** Uncertainty from significant digits in treatment planning system (TPS) and linac angular readout

Angular uncertainties—calculated at 8.02 cm off‐axis	Magnitude (mm)
Position from TPS angle calculation (all angles combined)	0.12
Position from linac angle readout (all angles combined)	0.12
**All sources summed in quadrature =**	**0.17**

An additional source of error is the physicist's ability to place the phantom in the correct position and orientation. To some extent, this error can be mitigated by examining the results of the off‐axis Winston–Lutz (OAWL) images while they are acquired on the linac. Often, setup errors can be readily noticed. This setup error is complicated by the additional requirement that, unlike an isocentric W–L, deviations in angular orientation must be minimized to the greatest extent possible. That said, a few extra minutes of care during setup, as compared to an isocentric W–L, seems to be enough to mostly overcome this additional complication. This observation is supported by the favorable agreement between our off‐axis results and our isocentric results.

## Appendix C

An excel spreadsheet named “OAWL Calculator.xlsx,” using this proposed OAWL methodology, can be found at the following link: https://github.com/eagleanton/OAWL‐files.

This file is presented for demonstration purposes only. A qualified medical physicist should verify all field parameters used for OAWL testing before clinical use.
